# Supplementary material for: Low-Salt Diet Regulates the Metabolic and Signal Transduction Genomic Fabrics, and Remodels the Cardiac Normal and Chronic Pathological Pathways
Source: Curr Issues Mol Biol. 2024 Mar 12;46(3):2355–85. doi: 10.3390/cimb46030150 (PMC10969083; doi:10.3390/cimb46030150)
Supplement: Supplementary file 1 [file cimb-46-00150-s001.zip › cimb-2887816-supplementary.pdf]

current issues in molecular biology

MDPI

Article

Low-salt Diet Regulates the Metabolic and Signal Transduction Genomic Fabrics, and Remodels the Cardiac Normal and Chronic Pathological Pathways

Dumitru A. Iacobas [1]*, Haile Allen [1] and Sanda Iacobas [2]

Supplementary Materials

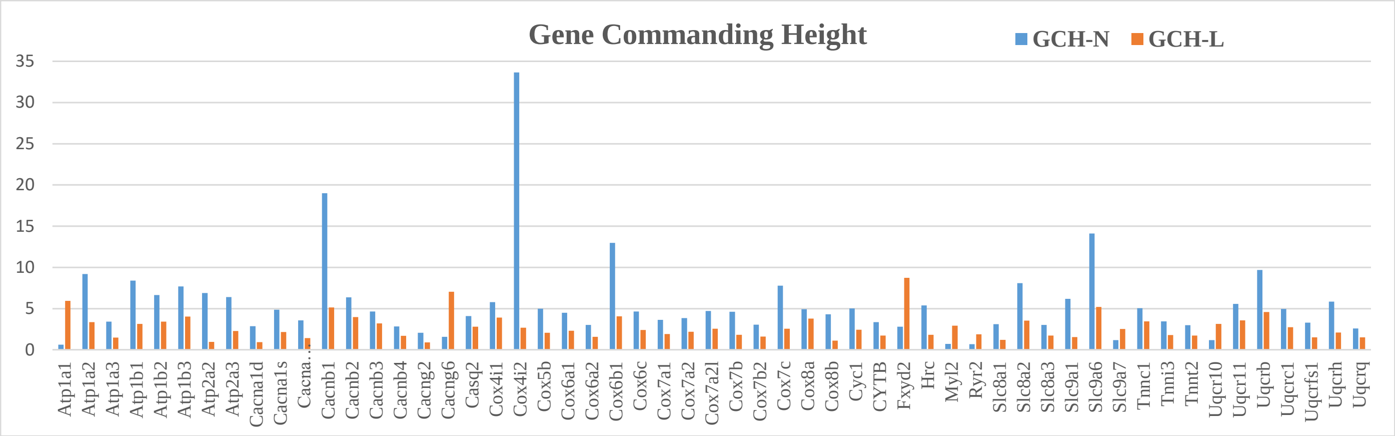

Figure S1: Gene Commanding Heights (GCH) within the KEGG-constructed CMC (Cardiac Muscle Contraction) pathway [51]. Note the reduction of the GCH scores for most CMC genes in LSD.

Citation: To be added by editorial staff during production.

Academic Editor: Firstname Lastname

Received: date
Revised: date
Accepted: date
Published: date

Copyright: © 2024 by the authors. Submitted for possible open access publication under the terms and conditions of the Creative Commons Attribution (CC BY) license (https://creativecommons.org/licenses/by/4.0/).

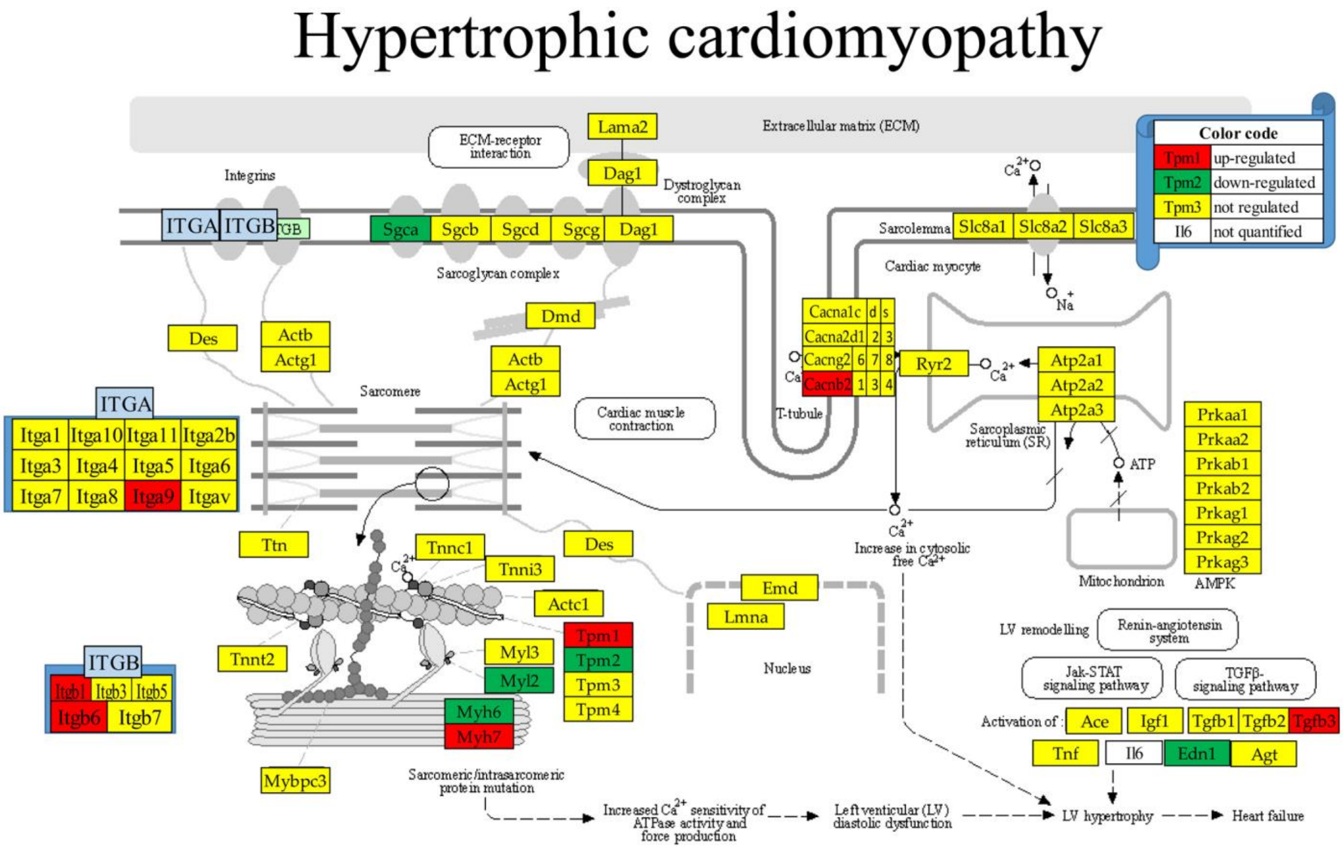

Figure S2: Regulated genes in the KEGG-constructed pathway Hypertrophic cardiomyopathy. Regulated genes: *Cacnb2* (calcium channel, voltage-dependent, beta 2 subunit), *Edn1* (endothelin 1), *Itga9* (integrin alpha 9), *Itgb1* (integrin beta 1), *Itgb6* (integrin beta 6), *Myh6/7* (myosin, heavy polypeptide 6, cardiac muscle, alpha/7, cardiac muscle, beta), *Myl2/4* (myosin, light polypeptide 2/4), *Sgca* (sarcoglycan, alpha (dystrophin-associated glycoprotein)), *Tgfb3* (transforming growth factor, beta 3), *Tpm1/2* (tropomyosin 1 alpha/2 beta).
